# Laparoscopic repair of paraesophageal hiatus hernia in infancy

**DOI:** 10.4103/0971-9261.44766

**Published:** 2008

**Authors:** Anjani Kumar Kundal, Noor Ullah Zargar, Anurag Krishna

**Affiliations:** Department of Pediatric Surgery, Sir Ganga Ram Hospital, Delhi - 110 060, India

**Keywords:** Diaphragmatic hernia, paraesophageal hiatus hernia, recurrent chest infections

## Abstract

Paraesophageal hiatus hernia (PEHH) is an uncommon type of diaphragmatic hernia in the pediatric age group. Two patients aged 5-months and 8-months presented with respiratory symptoms and underwent a laparoscopic repair. Preoperative assessment included chest x-ray and CT scan. We suggest that laparoscopic repair of PEHH in infants is safe and preferred mode of the treatment.

## INTRODUCTION

Paraesophageal hiatal hernia (PEHH) is an uncommon type of diaphragmatic hernia in the pediatric age group.[1] These hernias may be detected incidentally or may present with recurrent chest infections or vague gastrointestinal symptoms. Being rare and presentation with non-specific clinical features, diagnosis is often delayed till adulthood.[2] The basic principle of the surgical repair is to reduce the width of the diaphragmatic defect. Traditionally, this has been done by an open operation. More recently, laparoscopic repair is the preferred method of treatment in adults.[3–5] We report two infants who underwent successful laparoscopic repair.

## PATIENTS AND METHODS

Two girl infants, aged 5-months and 8-months presented with recurrent chest infections starting from early.

**Figure 1:**
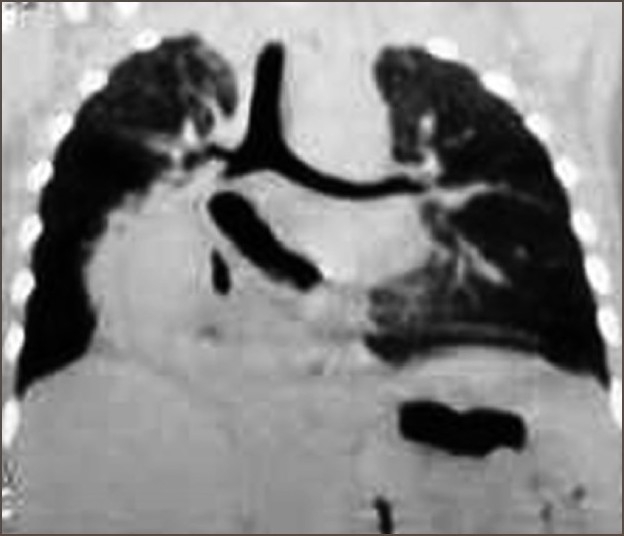
Coronal reconstruction of CT scan showing contrast filled stomach in the posterior mediastinum on right side of the chest

In both cases; the chest x-ray showed the stomach lying in the right side of the chest. The CT scan showed a hiatus hernia with herniation of the entire stomach into the right lower thorax [Figure 1]. Anesthetic induction was with sevofluorane, oxygen and air (FiO2-0.4). Endotracheal intubation was achieved with vecuronium. Fentanyl was used for analgesia. Routine monitoring were carried out using pulse oximeter, ECG, precordial stethoscope, automated blood pressure, EtCO2, and temperature along with peak inspiratory pressure (PIP). Carboperitoneum was achieved at 10 mm Hg at a CO2 flow rate of 2.5 L/min. All parameters were recorded prior to insufflation, after carboperitoneum, change of position (reverse Trendelenburg) and after desufflation. Ports were made. Stomach was seen herniating, which was reduced back in to the abdomen. The hernial sac was identified, dissected and excised. The right and left crus of the diaphragm were cleared. Lower oesophagus was mobilized partially. Large hiatal opening was closed around the esophagus with 2-0 silk interrupted sutures. In case 1, the fundus of the stomach was hitched to the left cupola of the diaphragm.

## RESULTS

The intra-operative period was uneventful. Mean operative time was 140 min (150,130 min). There was significant change in mean arterial pressure (MAP) and heart rate, which restored to normal soon after desufflation. These hemodynamic and respiratory alterations at intra-abdominal pressure (IAP) of 10 mm Hg and reverse Trendelenburg were well tolerated in both the patients. No blood transfusion was required. The postoperative recovery was uneventful. The infants were fed within 24 h of surgery and were discharged from the hospital at 48 h. At 3-months follow up, both children were well.

## DISCUSSION

Congenital PEHH occurs because of absent or abnormally lax anatomic anchors of the stomach, gastroesophageal junction, as well as a paraesophageal defect.[2] The fundus of the stomach is pushed into the chest by positive IAP and pulled up by the negative intra-thoracic pressure. If herniation progresses, the entire fundus and proximal antrum may migrate into the thorax, and organoaxial volvulus may occur.[6,7] This can result in obstruction at the level of the cardia and/or pylorus, leading to gastric or esophageal dilatation with mediastinal shift.[6,8] As the hiatus enlarges, bowel and omentum may also herniate. The presentation of PEHH may be acute or chronic. The acute presentation may be with life-threatening complications such as incarceration, obstruction, gangrene, perforation, bleeding, anemia, as well as acute respiratory complications.[6,8,9] They are easy to diagnose. In the chronic mode, repeated chest infections occur, presenting with fever and attacks of coughing. Intermittent vomiting may be present and commonly it may be attributed to the reflux disease common in this age group. Irritability and refusal to eat may be the other symptoms. On chest x-ray, typical findings include the presence of abdominal organs in the thorax, demonstrated by the extraneous densities or gas bubbles or by the presence of the nasogastric tube in the thorax. Opacity in the cardiophrenic angle may be the only clue to diagnosis in many cases. CT scan establishes a definitive diagnosis and defines the herniated contents and their nature more accurately. Routine elective repair is recommended in infants with congenital PEHH.[2] The principle of surgical repair includes resection of the sac, closure of the hiatal defect, and an antireflux or fixation procedure. It is suggested that hernial sac should be excised to allow sufficient closure of the hiatus, thereby reducing the risk of recurrence.[10] Other authors do not routinely excise the sac because of potential risk of pericardial, pleural, and mediastinal injuries.[11] Narrowing of the hiatus by approximating the two crura using nonabsorbable suture is the most important part of the repair.[12,13] Not all surgeons recommend adding an antireflux procedure to the hiatal repair. In infants, the esophagus is not short and thus complete sac excision and then adeqate esophageal mobilization are sufficient in satisfactory anatomic repair. There may be reasons to consider adding fundoplication to the repair of the hernia.[7]

Laparoscopic repair of PEHH is the preferred surgical technique in adults.[3–5] Reduction of the sac content and closure of the hiatal defect can be easily carried out with minimal surgical trauma and rapid recovery of the patient.
